# Toward Biological Pacing by Cellular Delivery of Hcn2/SkM1

**DOI:** 10.3389/fphys.2020.588679

**Published:** 2021-01-06

**Authors:** Anna M. D. Végh, Arie O. Verkerk, Lucía Cócera Ortega, Jianan Wang, Dirk Geerts, Mischa Klerk, Kirsten Lodder, Ruby Nobel, Anke J. Tijsen, Harsha D. Devalla, Vincent M. Christoffels, Max Medina-Ramírez, Anke M. Smits, Hanno L. Tan, Ronald Wilders, Marie José T. H. Goumans, Gerard J. J. Boink

**Affiliations:** ^1^Department of Cell and Chemical Biology, Leiden University Medical Center, Leiden, Netherlands; ^2^Department of Medical Biology, Amsterdam Cardiovascular Sciences, Amsterdam UMC, University of Amsterdam, Amsterdam, Netherlands; ^3^Department of Experimental Cardiology, Amsterdam Cardiovascular Sciences, Amsterdam UMC, University of Amsterdam, Amsterdam, Netherlands; ^4^Netherlands Heart Institute, Utrecht, Netherlands; ^5^Department of Clinical Cardiology, Heart Center, Amsterdam Cardiovascular Sciences, Amsterdam UMC, University of Amsterdam, Amsterdam, Netherlands

**Keywords:** biological pacemaker, gene therapy, cell therapy, progenitor cells, HCN channels, SkM1 channels

## Abstract

Electronic pacemakers still face major shortcomings that are largely intrinsic to their hardware-based design. Radical improvements can potentially be generated by gene or cell therapy-based biological pacemakers. Our previous work identified adenoviral gene transfer of Hcn2 and SkM1, encoding a “funny current” and skeletal fast sodium current, respectively, as a potent combination to induce short-term biological pacing in dogs with atrioventricular block. To achieve long-term biological pacemaker activity, alternative delivery platforms need to be explored and optimized. The aim of the present study was therefore to investigate the functional delivery of Hcn2/SkM1 via human cardiomyocyte progenitor cells (CPCs). Nucleofection of Hcn2 and SkM1 in CPCs was optimized and gene transfer was determined for Hcn2 and SkM1 *in vitro*. The modified CPCs were analyzed using patch-clamp for validation and characterization of functional transgene expression. In addition, biophysical properties of Hcn2 and SkM1 were further investigated in lentivirally transduced CPCs by patch-clamp analysis. To compare both modification methods *in vivo*, CPCs were nucleofected or lentivirally transduced with GFP and injected in the left ventricle of male NOD-SCID mice. After 1 week, hearts were collected and analyzed for GFP expression and cell engraftment. Subsequent functional studies were carried out by computational modeling. Both nucleofection and lentiviral transduction of CPCs resulted in functional gene transfer of Hcn2 and SkM1 channels. However, lentiviral transduction was more efficient than nucleofection-mediated gene transfer and the virally transduced cells survived better *in vivo*. These data support future use of lentiviral transduction over nucleofection, concerning CPC-based cardiac gene delivery. Detailed patch-clamp studies revealed Hcn2 and Skm1 current kinetics within the range of previously reported values of other cell systems. Finally, computational modeling indicated that CPC-mediated delivery of Hcn2/SkM1 can generate stable pacemaker function in human ventricular myocytes. These modeling studies further illustrated that SkM1 plays an essential role in the final stage of diastolic depolarization, thereby enhancing biological pacemaker functioning delivered by Hcn2. Altogether these studies support further development of CPC-mediated delivery of Hcn2/SkM1 and functional testing in bradycardia models.

## Introduction

At present, electronic pacemaker therapy is the standard of care for patients with heart block and/or sinus node dysfunction. Despite the life-saving success of this therapy and more than 60 years of research and development, electronic pacemakers still have major shortcomings, such as inadequate sensitivity to autonomic modulation, suboptimal cardiac output, and a limited battery life time that requires surgical replacement every 8–10 years. Moreover, electronic pacemakers are prone to other hardware-related issues such as lead dislodgement or fracture, magnetic interference, and potentially lethal device-related infections (Rosen et al., 2004; van Rees et al., 2011; Kotsakou et al., 2015).

With the development of biological pacemakers, the ultimate goal is to overcome these hardware-related issues and provide a more physiologic mode of pacing that generates optimal cardiac output and is directly sensitive to autonomic modulation. Short-term proof-of-concept studies have demonstrated that this is feasible either via direct gene transfer or via transplantation of stem cells. With the latter approach, cells are either coaxed into a lineage of pacemaker cells, or are merely used as a vehicle for delivery of ion channel function, following *ex vivo* gene transfer. The approach of *ex vivo* gene transfer to stem cells and subsequent transplantation has extensively been studied with human mesenchymal stem cells (hMSCs) (Potapova et al., 2004; Plotnikov et al., 2007; Jun et al., 2012; Lu et al., 2013). These studies evaluated pacing properties of Hcn2 overexpressing hMSCs that were transplanted into the hearts of dogs with complete heart block and showed a degree of function that was largely similar to adenoviral gene transfer. Yet unfortunately, function deteriorated after 6–8 weeks post-transplantation and was thus not sustained much beyond the expression window of adenoviral gene transfer (i.e., 2–4 weeks) (Kass-Eisler et al., 1993; Tripathy et al., 1996). To explore potential alternative cell sources that can provide function over a much longer follow-up period we have previously explored the use of human cardiomyocyte progenitor cells (CPCs). These CPCs have previously been shown to reside in the mouse heart for at least 12 weeks after transplantation and are able to functionally couple to mouse cardiomyocytes, without manifesting unwanted proliferation or differentiation beyond the cardiac lineage (Smits et al., 2009a; Végh et al., 2019). In contrast to MSCs, transplanted CPCs appear not to migrate away from the injection site, enhancing their potential as biological pacemakers by generating pacemaker activity from a more localized position (Gyöngyösi et al., 2008; Smits et al., 2009a; Bruzauskaite et al., 2016). Moreover, *in vitro* studies have demonstrated that CPCs can function as an effective vehicle for HCN-based biological pacing (Végh et al., 2019).

Over the past decade, several alternative gene transfer strategies have been explored to optimize HCN-based biological pacing. Overexpression of alternative HCN isoforms (i.e., HCN1, HCN2, and HCN4) and HCN2-based variants have been explored, but these resulted in only minor improvement (e.g., mHcn2-E324A) or, in marked contrast, lead to excessive outcomes with recurrent ventricular tachycardia in case of the Hcn2/Hcn1 chimera Hcn212 (Bucchi et al., 2006; Plotnikov et al., 2008; Shlapakova et al., 2010; Boink et al., 2012b, 2013). On the other hand, substantial improvements in baseline and maximal beating rates have been obtained by combined overexpression of Hcn2 and the skeletal muscle sodium channel SkM1. This previous study showed that combined overexpression of Hcn2 with SkM1 improved pacemaker function compared to Hcn2 alone, by hyperpolarization of the action potential threshold (Boink et al., 2013). In an effort to engineer a long-term biological pacemaker, the present study therefore investigated cellular delivery of Hcn2/SkM1 and further explored the mechanism of action of this transgene combination.

## Materials and Methods

### Cell Culture

Human fetal hearts were obtained after elective abortion under informed consent, with approval of the Medical Ethics Committee of the Leiden University Medical Center (number P08.087). The research was performed in accordance with the principles of the Declaration of Helsinki. Human CPCs were isolated from these hearts using magnetic activated cell sorting (MACS) as previously described (Smits et al., 2009b). In short, second trimester hearts were collected, and a single cell suspension was obtained by cutting the heart in small pieces and digesting the pieces with 500 ng/mL collagenase-A (Roche, 10103578001). Single cells were incubated with anti-Sca-1-FITC antibody [Miltenyi Biotec, Anti-Sca-1 MicroBead Kit (FITC), 130-092-529, 1:10 in M-buffer], and subsequently with anti-FITC microbeads [Miltenyi Biotec, Anti-Sca-1 MicroBead Kit (FITC), 130-092-529 1:10 in M-buffer]. Cells bound to the microbeads were isolated using a MiniMACS separation column (Miltenyi Biotec, type MS+, 130-042-201) and taken in culture in 0.1% gelatin coated wells with SP++ medium: EGM-2 (Lonza, CC-3162) with M199 (Gibco, 31150-030) in a 1:3 mixture, supplemented with 10% FBS and 1% non-essential amino acids (Gibco, 11140-035) complemented with 10 ng/mL basic fibroblast growth factor (bFGF, Sigma F0291). Cells were cultured at 37°C and 5% CO_2_ in SP++ medium and passaged at a confluency of ≈80% using trypsin (Gibco, 25200-056).

### Transduction and Nucleofection of CPCs

#### Nucleofection

Cardiomyocyte progenitor cells were nucleofected using the Lonza Amaxa 2b Nucleofector Device and the Human Stem Cell Nucleofector I Kit according to manufacturers’ protocol. For protocol optimization, GFP was nucleofected using the pmaxGFP plasmid (Amaxa, Cologne, Germany). Briefly, 1 × 10^6^ cells were resuspended in 100 μL of nucleofection buffer and 2 μg of pmaxGFP. To determine the optimal nucleofection condition, several programs were tested. Each program differs in duration and intensity of the pulsation as described by the manufacturer. After nucleofection, cells were seeded in six well plates with SP++ medium. Program X001 was selected and used in further nucleofections. For the comparison of GFP expression with or without Woodchuck Hepatitis Virus Posttranscriptional Regulatory Element (WPRE), and for FACS measurements, cells were nucleofected with 1 μg of plasmid.

For the optimization of the construct, 0.5 μg pmaxGFP, 2 μg mouse(m)Hcn2-IRES-DsRed, and 2 μg rat(r)SkM1-IRES-GFP plasmid was used. For the combination of mHcn2 and rSkM1, 2 μg of each plasmid was co-nucleofected in the CPCs. Four days after nucleofection, cells were seeded on coverslips for immunocytochemistry.

For patch-clamp experiments, 2 μg of mHcn2 or 2 μg of rSkM1 plasmid (both without reporter markers) was co-nucleofected with 0.5 μg of GFP plasmid.

#### Transduction

For patch-clamp experiments, CPCs were transduced with VSVg pseudotyped LV-CMV-mHcn2-P2a-GFP-WPRE or LV-CMV-rSkM1-P2a-GFP-WPRE at an MOI of 5.0 and used after 2 days in culture. For the *in vivo* experiments, CPCs were transduced with VSVg pseudotyped LV-CMV-GFP-HPRE at an MOI of 5.0 and were used for transplantation experiments after two passages.

### Immunocytochemistry

Cells were fixed in 4% paraformaldehyde for 10 min at room temperature and washed 3 times in PBS. Cells were permeabilized with 0.1% Triton X-100 for 8 min and incubated overnight with the following antibodies diluted in 4% goat serum: mouse α-SkM1 antibody (Sigma-Aldrich, S9568, 1: 200) and rabbit α-HCN2 antibody (Alomone, APC-030, 1:200). The next day, cells were washed three times in PBS with 0.05% Tween 20, and incubated for 1 h at room temperature with the following secondary antibodies: goat- α-mouse-488 (Invitrogen, A-11001, 1:250) or goat α-rabbit-568 (Invitrogen, A-11011, 1:250) in PBS with 4% goat serum. Cells were washed three times in PBS with 0.05% Tween 20. Nuclei were counterstained with DAPI (1:5000) for 5 min and mounted in Mowiol (Sigma, 81381).

### Patch-Clamp Experiments

#### Data Acquisition

The Hcn2 and SkM1 currents (*I*_Hcn__2_ and *I*_SkM__1_, respectively) were measured in the whole-cell configuration of the patch-clamp technique using an Axopatch 200B amplifier (Molecular Devices Corporation, Sunnyvale, CA, United States). CPCs were harvested, stored in SP++ medium at room temperature (20°C), and studied within 4 h. Cell suspensions were put into a recording chamber on the stage of an inverted microscope (Nikon Diaphot), and single CPCs that visibly formed branches with the bottom of the recording chamber and exhibited green fluorescence were selected for electrophysiological measurements. Voltage control, data acquisition, and analysis were accomplished using custom software. Potentials were corrected for the estimated change in liquid-junction potential (Barry and Lynch, 1991). Signals were low-pass filtered with a cut-off frequency of 5 kHz and digitized at 5 and 20 kHz for *I*_Hcn__2_ and *I*_SkM__1_, respectively. Series resistance was compensated by ≥80%. Cell membrane capacitance (C_m_) was calculated by dividing the time constant of the decay of the capacitive transient after a −5 mV voltage step from −40 mV by the series resistance, and amounted to 24.8 ± 2.5 pF (*n* = 18).

#### Hcn2 Current

*I*_Hcn__2_ was recorded using the amphotericin-perforated patch-clamp technique at 36 ± 0.2°C. Bath solution contained (in mM): NaCl 140, KCl 5.4, CaCl_2_ 1.8, MgCl_2_ 1.0, glucose 5.5, HEPES 5.0; pH 7.4 (NaOH). Laboratory-made pipettes [2.5–3.5 MΩ; borosilicate glass (Harvard Apparatus, United Kingdom)] were filled with solution containing (in mM): K-gluc 125, KCl 20, NaCl 10, amphotericin-B 0.44, HEPES 5; pH 7.2 (KOH). In general, *I*_Hcn__2_ was measured during 6-s hyperpolarizing voltage clamp steps (test potentials ranging from −30 to −140 mV) from a holding potential of −30 mV. Next, a 6-s step to −120 mV was applied to record tail current followed by a 1-s pulse to 10 mV to ensure full deactivation (see Figure 5A for protocol; cycle length 18-s). In the experiments presented in Figure 3, depolarizing steps were limited up to −120 mV and were followed by an 8-s step to −120 mV for recording of tail currents. Activation kinetics were measured during the 6-s hyperpolarizing steps. The current-voltage (I–V) relation was constructed from the current measured at the end of the 6-s hyperpolarizing steps. Currents were normalized to C_m_. Tail current, plotted against test voltage, provided the activation-voltage relationship. The activation-voltage relation was normalized by maximum conductance and fitted with the Boltzmann function I/Imax = A/{1.0 + exp[(V– V_1__/__2_)/k]} to determine the half-maximum activation voltage (V_1__/__2_) and slope factor (k). Deactivation kinetics and reversal potential (E_rev_) were measured during depolarizing voltage clamp steps (test potentials ranging from −80 to −10 mV, duration 6 s) after a 5-s prepulse to −120 mV to ensure full activation (see Figure 5D for protocol; cycle length 15 s). The time course of *I*_Hcn__2_ (de)activation was fitted by the mono-exponential equation I/Imax = A × [1 – exp(−t/τ)], ignoring the variable initial delay in Hcn2 (de)activation (Végh et al., 2019), to determine the time constant of (de)activation τ.

#### SkM1 Current

*I*_SkM__1_ was measured at room temperature using the ruptured patch-clamp technique with patch pipettes (2.0–2.5 MΩ) containing (in mM): CsCl 10, CsF 110, NaF 10, EGTA 11, CaCl_2_ 1.0, MgCl_2_ 1.0, Na_2_ATP 2.0, HEPES 10, pH 7.2 (CsOH). Bath solution contained (in mM): NaCl 140, CsCl 10, CaCl_2_ 1.8, MgCl_2_ 1.0, glucose 5.5, HEPES 5.0, pH 7.4 (NaOH). The current density of the peak of *I*_SkM__1_ and voltage dependence of (in)activation were determined using the voltage protocols as shown in Figure 6. The holding potential was −120 mV (except when mentioned otherwise) and the voltage clamp steps were applied with a 5 s cycle length. Peak *I*_SkM__1_ was defined as the difference between peak and steady-state current. Current density was calculated by dividing the measured currents by C_m_. To determine the activation characteristics of *I*_SkM__1_, I–V curves were corrected for differences in driving force and normalized to maximum peak current. The time course of *I*_SkM__1_ inactivation and steady-state (in)activation curves were fitted using the above-mentioned mono-exponential and Boltzmann equations, respectively.

### *In vivo* Experiments

All mouse experiments were performed in accordance with the Guide for the Care and Use of Laboratory Animals and the protocols were approved by the Animal Ethics Committee of Leiden University Medical Center. Male immunocompromised NOD-SCID mice were chosen to minimize immune rejection against the transplanted cells. Mice aged 3–6 months were anesthetized with 4% isoflurane, intubated and maintained under 1.5–2% isoflurane during the procedure. Mice were given 0.05 mg/kg Buprenorphine prior to and 8–12 h after the procedure. After thoracotomy, 10 μL PBS containing ≈500.000 CPCs was divided over two injection-sites in the left ventricle near the left anterior descending coronary artery. Mice were randomly assigned to one of the three groups: untreated CPCs (*n* = 4), LV-GFP transduced CPCs (CPC LV-GFP) (*n* = 4) or GFP nucleofected CPCs (CPC NF-GFP) (*n* = 5). Seven days after the procedure, mice were sacrificed and hearts were collected. All procedures and injections were performed by a blinded investigator. The hearts were flushed with ice-cold PBS, fixed in 4% ice-cold paraformaldehyde, dehydrated with 30% sucrose overnight and embedded in Tissue-Tek O.C.T. compound (Sakura, 4583). After snap-freezing, the hearts were stored at −80°C, before they were cryosectioned. Whole mouse hearts were cryosectioned at 7 μm thickness from apex to base, and divided in 10 fractions of five sections each with equal distance (60 sections apart) from each other for immunostaining. In total, 50 sections throughout each heart were stained and analyzed.

### Immunohistochemistry

Sections were incubated overnight with the following primary antibodies in 1% bovine serum albumin (Sigma, 9048-46-8) in PBS with 0.1% Tween 20: chicken α-GFP (Abcam, ab13970, 1:200), human-specific rabbit α-Collagen-1 (Abcam, ab138492, 1:200), and mouse α-β1-integrin (Santa Cruz, SC-53711, 1:500). The next day, sections were washed and incubated with the following secondary antibodies: goat α-chicken IgG-488 (Life Technologies, A11039, 1:250), donkey α-rabbit IgG-647 (Life Technologies, A31573, 1:250), and donkey α-mouse IgG-555 (Life Technologies, A31570, 1:250).

### Computer Model

Functional effects of the electrical coupling of a human cardiomyocyte to CPCs expressing Hcn2 and/or SkM1 channels were assessed by computer simulations using the human ventricular cell model by Ten Tusscher et al. (2004), as updated by Ten Tusscher and Panfilov (2006), with its strong inward rectifier K^+^ current (*I*_K__1_) downscaled by 65%, based on patch-clamp data obtained from single ventricular cardiomyocytes that were enzymatically dissociated from non-diseased human donor hearts (Jost et al., 2013). Coupling of the model cell to a single CPC was implemented as a gap junctional conductance of 10 nS, as observed between heterologous cell pairs, such as hMSCs and myocytes (Valiunas et al., 2009). The membrane capacitance of a single CPC was set to 24.8 pF, in line with the aforementioned data. The Hcn2 current as expressed in a single CPC was included as *I*_Hcn__2_ = y × g_Hcn__2_ × (V_m_ – E_Hcn__2_), in which V_m_ denotes membrane potential and the fully activated conductance g_Hcn__2_ and reversal potential E_Hcn__2_ amount to 0.4 nS/pF and −39.8 mV, respectively, as observed in our patch-clamp experiments. The kinetics of the gating variable y were determined by the steady-state activation curve and time constant of (de)activation, as obtained in these experiments. The equations for the *I*_SkM__1_ of the CPC were identical to those for the sodium current (*I*_Na_) of the ventricular cell model, but with a +10.6 mV shift in steady-state activation as well as a +18.8 mV shift in steady-state inactivation, based on our patch-clamp data on *I*_SkM__1_. The fully activated conductance of *I*_SkM__1_ was set to 7.0 nS/pF, estimated from our experimental observations, corrected for temperature.

### Statistical Analysis

For the *in vivo* expression, of β1-integrin and Collagen-1, non-parametric Kruskal-Wallis tests were carried out, followed by Dunn’s test for multiple comparisons. Since no GFP expression was measured in the untreated CPC group, statistics in this experiment were carried out by performing a non-parametric Mann–Whitney test between transduced and nucleofected CPCs. All data are presented as mean ± SEM. *P* < 0.05 was considered statistically significant.

## Results

### Nucleofection of CPCs Is Improved by the Addition of a WPRE-Element

**FIGURE 1:**
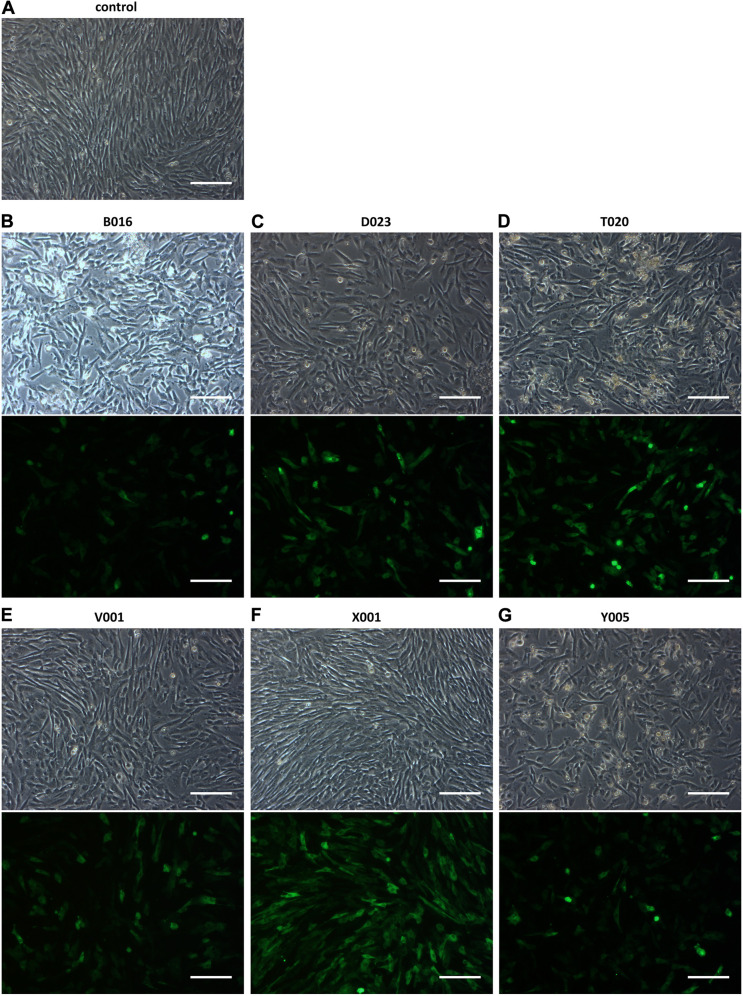
Nucleofection optimization of CPCs with different programs. **(A)** Control figure shows nucleofected CPCs with no DNA. **(B–G)** GFP expression in CPCs as a result of different nucleofection programs. Upper rows show bright field images of nucleofected CPCs and lower rows show GFP expression. Images shown are representative examples of duplicate experiments. Scale bars represent 200 μM.

**FIGURE 2:**
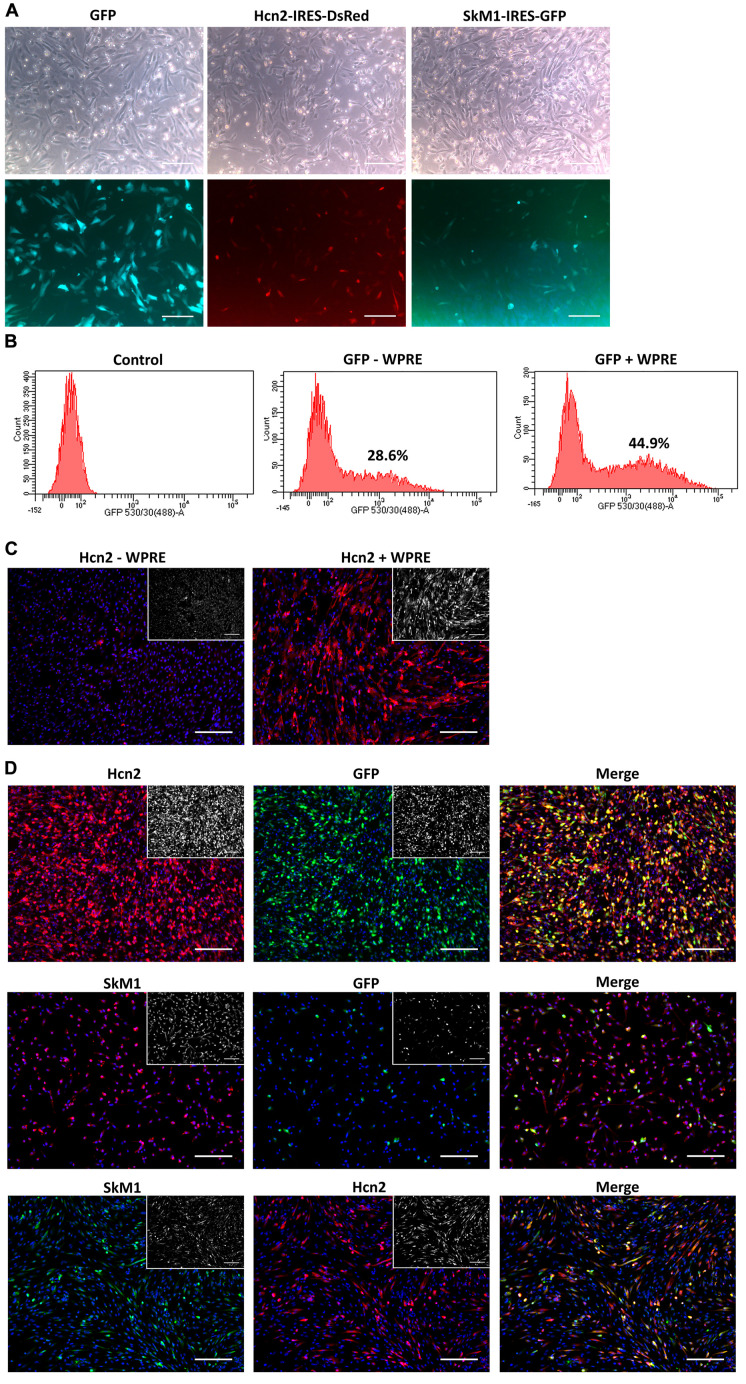
Nucleofection optimization of CPCs. **(A)** Nucleofection with GFP (left panels), Hcn2 (middle panels) or SkM1 (right panels) plasmids with IRES driven reporter markers. Upper panels show bright field images of nucleofected CPCs and lower panels show expression of reporter markers (DsRed in red and GFP in green). Scale bars represent 200 μM. **(B)** FACS data of GFP expression in nucleofected CPCs with a GFP plasmid with (right panel) or without (middle panel) the WPRE motif. Control cells (left panel) do not express GFP. Percentages indicate the % GFP-positive counted cells. **(C)** Immunocytochemistry of nucleofected CPCs with an Hcn2 plasmid, without or with WPRE motif (left and right panel, respectively). Hcn2 is stained in red and nuclei are counterstained in blue. Staining without DAPI is shown in the insets. **(D)** Immunocytochemistry of co-expression of Hcn2 (upper panels) and SkM1 (middle panels) with GFP nucleofected CPCs in 4:1 ratio. Lower panels show co-expression of Hcn2 and SkM1 in 1:1 ratio. Staining without DAPI is shown in the insets. Scale bars represent 200 μM, *n* = 2.

To explore nucleofection as a gene delivery method in CPCs, different programs (as pre-defined by the manufacturer) were tested to evaluate gene transfer and survival, where program X001 turned out to be superior in terms of GFP expression and cell survival (Figure 1). Next, this nucleofection program was used for the delivery of the functional genes Hcn2 and SkM1. Plasmids containing IRES reporter markers (GFP for SkM1 and DsRed for Hcn2) were used to assess efficiency of transfection and identify transfected cells for further experiments. However, the efficiency of nucleofection of Hcn2 and SkM1 was rather low in comparison with gene transfer of GFP alone (Figure 2A). In order to improve gene expression, the Woodchuck Hepatitis Virus Posttranscriptional Regulatory Element (WPRE) was added, which should enhance stability of the mRNA and therefore improve expression in the setting of nucleofection. Indeed, GFP was higher expressed when a plasmid containing the WPRE motif was used (Figure 2B). This was also the case when this was applied to the expression of Hcn2 (Figure 2C). Furthermore, we were able to achieve high efficiency of co-expression of Hcn2 or SkM1 together with GFP, which allowed us to visualize the cells that were efficiently transfected (Figure 2D, top two rows). In addition, high co-expression of Hcn2 and SkM1 was achieved with no detrimental effect on cell viability.

**FIGURE 3:**
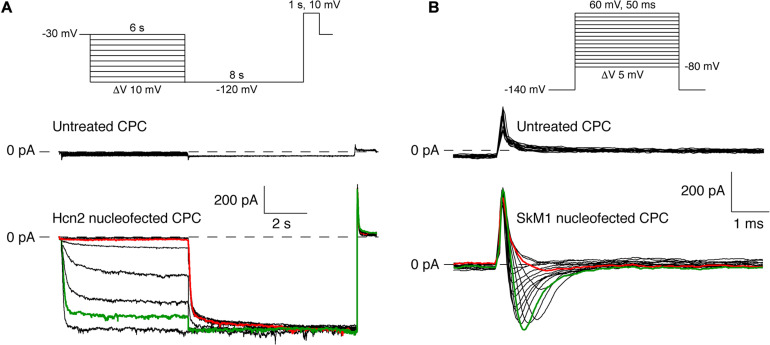
Pilot experiments of Hcn2 and SkM1 currents measured at 36°C and room temperature, respectively, in single nucleofected CPCs. **(A)** Top panel, protocol used. Middle panel, recording of an empty CPC. Bottom panel, Hcn2-encoded “funny current” (at test potentials ranging from –30 to –120 mV) in an Hcn2 nucleofected CPC. **(B)** Top panel, protocol used. Middle panel, recording of an empty CPC. Bottom panel, skeletal fast sodium current (at test potentials ranging from –50 to +20 mV) in a SkM1 nucleofected CPC. SkM1 was measured from a holding potential of –140 mV.

### Nucleofection of CPCs Results in Hcn2 and SkM1 Currents

Next, we assessed whether DNA transfer by nucleofection of the Hcn2 gene and SkM1 gene resulted in functional membrane currents. CPCs transduced with only GFP neither express currents upon hyperpolarization nor on depolarization (Figures 3A,B), which agrees with our previous study (Végh et al., 2019). Figure 3A, bottom panel, shows membrane currents in an Hcn2-nucleofected, GFP-positive CPC. Typical for an HCN-encoded “funny”-current (*I*_f_), an inward current was activated following hyperpolarizing steps from a holding potential of −30 mV, and this hyperpolarization-activated current became larger and activated more rapidly at increasingly negative potentials. In this particular example, activation threshold was around −70 mV (red trace) and full activation was reached at a potential of −110 mV (green trace). Figure 3B, bottom panel, shows membrane currents in an SkM1-nucleofected, GFP-positive CPC. Upon depolarizing voltage steps from −140 mV, a fast activating and inactivating inward current was activated, typical for a fast sodium current (*I*_Na_). In this particular example, the current activated at −40 mV (red trace), and was maximal at −20 mV (green trace).

Although nucleofection of the Hcn2 gene and SkM1 gene resulted in functional membrane currents, GFP expression was relatively low and difficult to detect by eye in the patch-clamp set-up. We therefore continued the patch-clamp experiments in lentivirally transduced CPCs which resulted in sufficient GFP expression in a relatively large number of CPCs.

### Biophysical Characterization of Lentivirally Transduced CPCs Expressing Hcn2 and SkM1

**FIGURE 4:**
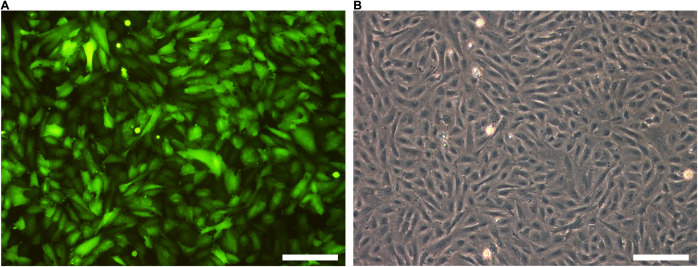
GFP is highly expressed after lentiviral transduction. **(A)** GFP expression of LV-GFP transduced CPCs. **(B)** Bright field picture of CPCs. Scale bars represent 100 μM, *n* = 5.

**FIGURE 5:**
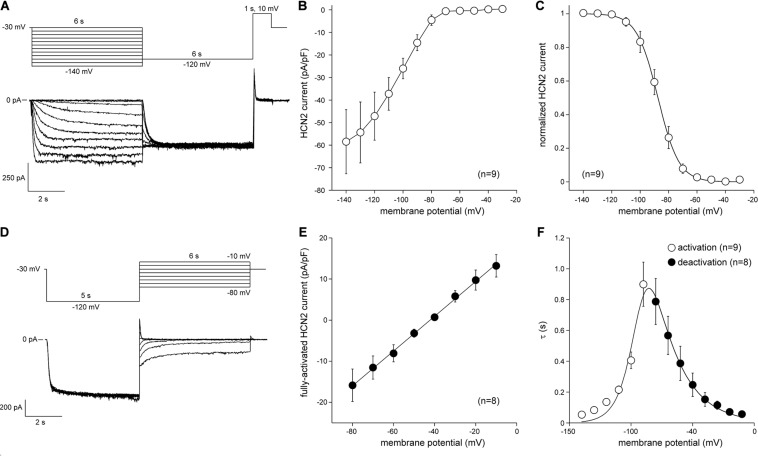
Hcn2 current measured at measured at 36°C in single LV-transduced CPCs. **(A)** Typical Hcn2 current traces (bottom panel) activated with a double pulse voltage clamp protocol (top panel) to determine current density and activation properties. **(B)** Average current-voltage (I–V) relationship of the Hcn2 current. **(C)** Voltage dependence of Hcn2 current activation. Solid line is the Boltzmann fit to the experimental data. **(D)** Typical Hcn2 current traces (bottom panel) activated with a double pulse voltage clamp protocol (top panel) to determine the reversal potentials and deactivation properties. **(E)** I–V relationship of the fully activated Hcn2 current. Solid line is the linear fit to the experimental data. **(F)** Time constants of (de)activation. Solid line is the best fit curve to the equation *τ* = 1/[A1 × exp(–V/B1) + A2 × exp(V/B2)], where *τ* is the activation or deactivation time constant (s), and A1, A2, B1, and B2 are calculated fitting parameters, which amount to 3.2164⋅10^– 8^s^– 1^, 9.2311 s^– 1^, 0.041248 mV, and 21.724 mV, respectively (Qu et al., 2004).

The next step was to transduce CPCs with lentiviral constructs. First, CPCs were transduced with LV-CMV-GFP-HPRE, which lead to high GFP expression *in vitro* (Figure 4). Then, CPCs were transduced with LV-CMV-mHcn2-P2a-GFP-WPRE or LV-CMV-rSkM1-P2a-GFP-WPRE to study the properties of the Hcn2- and SkM1-encoded currents using patch-clamp methodology. Figure 5A shows typical Hcn2 recordings from a single lentivirally transduced CPC. The average I–V relationship of *I*_Hcn__2_ is summarized in Figure 5B. CPCs expressing Hcn2 showed large time-dependent inward currents in response to the hyperpolarizing voltage steps, typical for Hcn2 (Figures 5A,B). To analyze voltage-dependence of Hcn2 activation, we plotted the normalized tail current amplitude against the preceding hyperpolarizing potential and fitted a Boltzmann function to the data. The average curve is shown in Figure 5C. The average V_1__/__2_ and k of the Boltzmann fit to the data were −81.4 ± 7.6 and −6.2 ± 0.8 mV (*n* = 9), respectively. The voltage-dependence of the fully activated current was evaluated over a large range of potentials (−80 to −10 mV) by measuring the tail current amplitudes after a hyperpolarizing pulse to −120 mV. Figure 5D shows a typical example, and Figure 5E shows the average I–V relationship of the fully activated *I*_Hcn__2_. The average reversal potential was −39.8 ± 1.1 mV (*n* = 8). Activation and deactivation time constants (Figure 5F) were obtained from mono-exponential fits of the step (Figure 5A) and tail (Figure 5D) currents, respectively.

**FIGURE 6:**
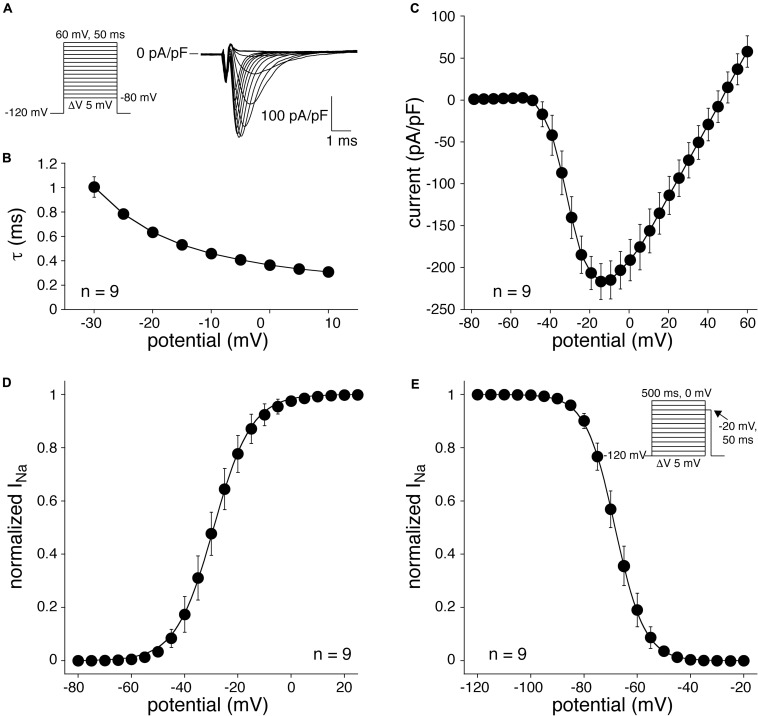
SkM1 current measured at room temperature in single CPCs. **(A)** Left: voltage clamp protocol used. Right: Typical example of SkM1 current in response to 50-ms depolarizing voltage steps ranging from –50 and +20 mV, from a holding potential of –120 mV. **(B)** Average time constants of fast and slow SkM1 current inactivation. **(C)** Average I–V relationship. **(D)** Average steady-state activation curve. The solid line is the Boltzmann fit to the average data. **(E)** Average steady-state inactivation curve. The solid line is the Boltzmann fit to the average data. Inset: voltage clamp protocol for determination of inactivation.

Figure 6A shows representative SkM1 currents in a lentivirally transduced CPC activated by 50-ms depolarizing voltage clamp steps of 5 mV increment. The current starts to activate around −50 mV, peaks around −10 mV, and subsequently decreases in amplitude due to the reduction in Na^+^ driving force. Figure 6B summarizes the average time constants of *I*_Na_ inactivation, which typically is faster at more depolarized potentials. Figure 6C shows average data for the I–V relationship. The average maximal current density was −217 ± 22 pA/pF (*n* = 9). Figure 6D shows the average voltage-dependency of activation. The average V_1__/__2_ and k of the voltage-dependency of activation were −27.3 ± 2.5 and 5.6 ± 0.5 mV (*n* = 9), respectively. The voltage-dependency of inactivation was measured using a two-pulse protocol where a 500-ms conditioning prepulse to membrane potentials between −120 and 20 mV, to induce steady-state inactivation, was followed by a 50-ms test pulse (Figure 6E, inset). The average V_1__/__2_ and k of the voltage-dependency of inactivation were −65.1 ± 1.3 and −4.6 ± 0.1 mV (*n* = 9), respectively.

### Lentiviral Transduced CPCs Survive Better *in vivo*

**FIGURE 7:**
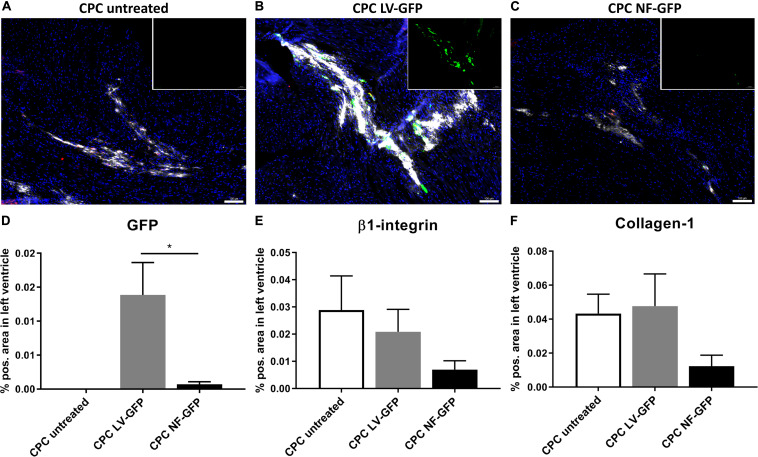
GFP expression and CPC engraftment 1 week after transplanting CPCs to the left ventricle near the left anterior descending artery. **(A–C)** Typical examples of expression of GFP (green), β1-integrin (red), and Collagen-1 (white) in mouse hearts injected with **(A)** unmodified CPCs, **(B)** LV-GFP transduced CPCs or **(C)** GFP nucleofected (NF) CPCs. Inset: GFP expression alone. Scale bars represent 100 μM. **(D–F)** Expression of GFP, β1-integrin, and Collagen-1 in mice injected with unmodified CPCs (*n* = 4), lentivirally transduced CPCs (*n* = 4) or nucleofected CPCs (*n* = 5), measured as% positive area of total left ventricular area. * indicates *P* < 0.05.

Encouraged by the initial *in vitro* experiments we proceeded with an *in vivo* comparison between GFP-nucleofected and GFP-lentivirally transduced cells. One week after transplantation, the presence of CPCs and GFP expression was determined in the mouse heart. GFP was highly expressed after transplantation of transduced CPCs, whereas the nucleofected CPC group showed almost no GFP expression after transplantation (Figures 7A–D). The human-specific β1-integrin was used as a measure for the presence of the human-derived CPCs after transplantation (Smits et al., 2009a). Since the same number of cells is transplanted in every group, β1-integrin expression can be used as a measure for survival of the cells at a certain time point. The analysis clearly indicated a trend toward lowest expression of β1-integrin in the nucleofection group (Figure 7E). Secretion of matrix protein human collagen-1 was also lower in nucleofected CPCs. Visualization of secreted collagen-1 allows for detection of human cell grafts even beyond cell death and is therefore a measure for cumulative human cell engraftment (Bax et al., 2019) (Figure 7F). Collectively, these data suggest both engraftment and survival are compromised with nucleofected cells compared to unmodified or lentivirally transduced CPCs. As a result, GFP gene delivery was also higher with lentivirally transduced CPCs. We therefore proceeded with computer modeling studies, based on the biophysical characterization obtained from lentivirally transduced cells.

### Contribution of SkM1 to Diastolic Depolarization

**FIGURE 8:**
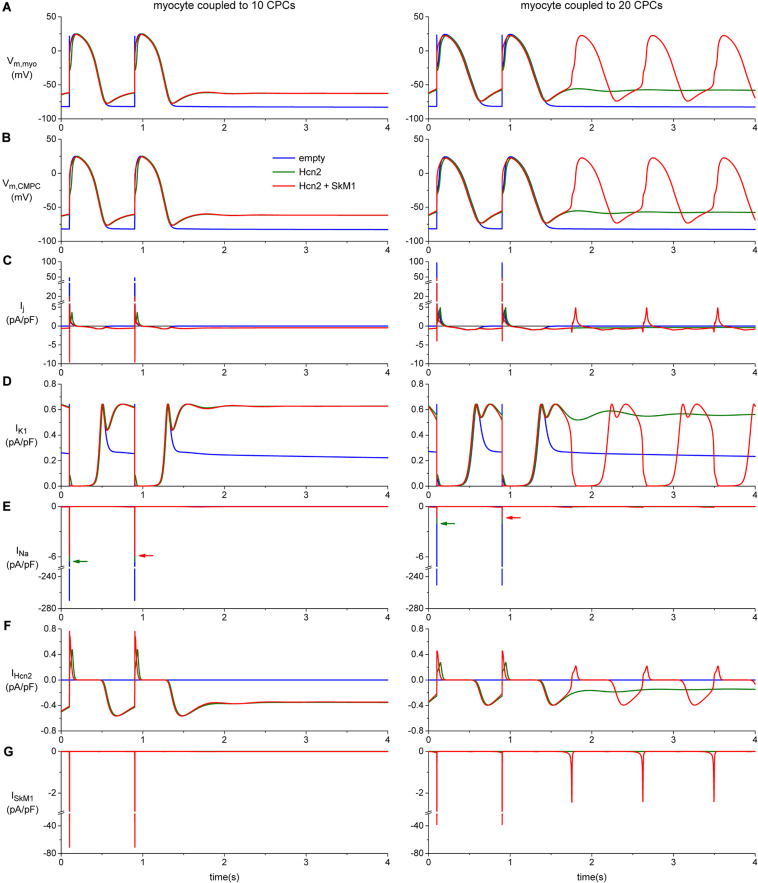
Computer simulation of action potentials of a human ventricular myocyte coupled to 10 (left panels) or 20 CPCs (right panels) overexpressing Hcn2 alone (green line), or Hcn2 + SkM1 (red line). The blue line shows the action potentials of the myocyte coupled to 10 (left) or 20 (right) empty CPCs. **(A)** Last two action potentials of a train of 100, induced by a 1 ms, ≈50% suprathreshold stimulus. Formation of stable pacemaker function after ending the train of stimuli is found only with the larger number of CPCs and only in the Hcn2 + SkM1 group and not in the simulation with Hcn2 alone. **(B)** Corresponding membrane potential of each of the CPCs. **(C)** Total junctional current flowing from the myocyte to the CPCs. Positive junctional current is outward for the myocyte, but inward for the CMPCs, and vice versa. **(D,E)** Membrane ion currents *I*_K__1_ and *I*_Na_ of the myocyte. **(F,G)** Membrane ion currents *I*_Hcn__2_ and *I*_SkM__1_ of each of the CPCs.

To investigate how the biophysical properties of Hcn2 and SkM1, as recorded in lentivirally transduced CPCs, would impact on action potential morphology and spontaneous activity we proceeded with computer simulation studies. These studies indicated that significant diastolic depolarization can be induced when a moderate number of CPCs is coupled to one ventricular working cardiomyocyte, as illustrated for 10 CPCs in the left panels of Figure 8. However, stable automaticity could only be obtained if at least 14 CPCs were coupled to the ventricular working cardiomyocyte. The mechanism underlying this stable automaticity is illustrated in the right panels of Figure 8 for a total of 20 CPCs.

These simulations with a total of 20 CPCs illustrated stable automaticity upon ending external stimulation exclusively with Hcn2/SkM1-expressing CPCs and not with Hcn2 alone (Figure 8, right panels). Interestingly, SkM1 contributed to the final stage of diastolic depolarization, supporting depolarization toward the voltage range of L-type Ca^2+^-channel activation. In this setting, mere Hcn2-mediated diastolic depolarization was insufficient to reach the threshold for Ca^2+^-channel opening.

The intriguing increase in action potential duration upon coupling the myocyte to 10 CPCs (Figure 8A, left), and even more so with 20 CPCs (Figure 8A, right), is the result of the junctional current (Figure 8C), which is inward to the myocyte during the repolarization phase of the action potential. The voltage changes of the CPCs closely follow those of the myocyte (Figures 8A,B), but during repolarization the myocyte is in the lead, with the CPCs lagging behind. As a consequence, the membrane potential of the CPCs is more positive than that of the myocyte during early repolarization and less negative during late repolarization, so that the junctional current acts as an inward current for the myocyte and slows its repolarization.

## Discussion

In the present study, we investigated cellular gene delivery of Hcn2 and SkM1 in an effort toward engineering long-term biological pacemaker activity. Although both nucleofection and lentiviral transduction were effective gene transfer tools to manipulate CPCs, lentiviral transduction was superior with regard to survival of gene-modified cells in the context of *in vivo* transplantation. Subsequent patch-clamp studies on lentivirally transduced Hcn2/SkM1-expressing cells demonstrated robust functional transgene expression that appeared sufficient to induce biological pacemaker activity in computer modeling studies, provided that *I*_K__1_ was substantially reduced as compared to the original model (Ten Tusscher et al., 2004; Ten Tusscher and Panfilov, 2006), based on more recent comprehensive patch-clamp data on the amplitude of *I*_K__1_ in human ventricular cardiomyocytes (Jost et al., 2013).

Both nucleofection and transduction of CPCs resulted in robust functional expression of Hcn2 and SkM1 *in vitro*. Previous studies have shown that lentiviral transduction or electroporation of MSCs was sufficiently effective to provide robust outcomes in terms of biological pacing and restoration of impulse propagation in the epicardial border zone (Potapova et al., 2004; Plotnikov et al., 2007; Jun et al., 2012; Lu et al., 2013). However, our results indicate that delivery of GFP *in vivo* is superior with lentivirally transduced cells. This appears primarily driven by higher gene-delivery efficiency *in vitro* and better survival of the lentivirally transduced CPCs after transplantation. Other studies that compare gene-transfer efficiency and *in vitro* survival of several (stem) cell types also showed results in favor of lentiviral transduction over electroporation/nucleofection (Dullaers et al., 2004; Mcmahon et al., 2006; Li and Lu, 2009; Cao et al., 2010).

Transplantation efficiency of CPCs showed moderate engraftment of unmodified and LV-transduced CPCs after 1 week. This was also found in a previous study, where CPC survival was around 3.5% 2 days after MI and transplantation of the cells (Smits et al., 2009a). In comparison, MSC survival was found to be less than 0.44% in SCID mice 4 days after myocardial infarction (MI) (Toma et al., 2002), and ≈1% in rats 24 h after MI (McGinley et al., 2013). Although engraftment rates were low in MI models, the effect on cardiac function of transplanting (cardiac) stem cells was still significant (Zwetsloot et al., 2016). One of the advantages over restoring damaged heart muscle after MI, is that the development of a biological pacemaker expectedly requires a lower degree of cell engraftment. In dog hearts, the minimum required amount of MSCs to create stable biological pacing was estimated to 700.000 cells, which adds to the feasibility of such a stem cell-based approach (Plotnikov et al., 2007).

The comparison between biological pacemaker strategies based on undifferentiated cells (i.e., CPCs and MSCs) and those that are based on pluripotent stem cells coaxed into a lineage of pacemaker cells remains difficult at this stage. The transplantation of human embryonic stem cell derived cardiomyocytes (ESC-CMs) in guinea pigs or pigs with atrioventricular (AV)-block resulted in ectopic activity at the site of transplantation (Kehat et al., 2004; Xue et al., 2005). However, difficulties in obtaining pure hESC-CM cultures, potential risk of teratoma formation and immunological issues have hampered further translational development of this technology for biological pacemaker applications. Human induced pluripotent stem cell-derived cardiomyocytes (hiPSC-CMs) represent a promising alternative given their potential use in autologous stem cell transplantation, thereby holding important promise to bypass immune rejection. Moreover, long term biological pacing has been encouraging as stable function was maintained throughout a 13 week canine study (Chauveau et al., 2017). Yet in terms of pacemaker function, further improvements appear still to be necessary, as beating rates were relatively low, and dependency on back-up pacing was still substantial (Chauveau et al., 2017). Ongoing efforts therefore focus on optimization of hiPSC differentiation, maturation and purification protocols (Protze et al., 2017). How these improvements will eventually compare to biological pacemakers that are made from undifferentiated cells, such as CPCs, remains to be determined by follow-up studies.

Detailed biophysical studies illustrate comparable, or even potentially more favorable kinetics for *I*_Hcn__2_ and *I*_SkM__1_ in lentivirally transduced CPCs vs. previously reported values in MSCs. The voltage of half-maximal activation of Hcn2 (−86 mV) falls well within the physiological range of cardiomyocyte membrane potentials and appears to be more positive than the value previously reported for Hcn2-expressing MSCs (−96 mV) (Potapova et al., 2004; Plotnikov et al., 2007). Moreover, the *I*_Hcn__2_ density also appears to be relatively high in CPCs compared to MSCs (58 pA/pF at −140 mV vs. 31 pA/pF at −150 mV). For SkM1, the comparison was slightly more complicated as the voltage of half-maximal inactivation was more negative (−65 mV) than the value reported in MSCs (−59 mV). On the other hand, the SkM1 peak current density was again much higher in CPCs than in MSCs (217 vs. 39 pA/pF) (Boink et al., 2012a). Yet, it remains difficult to draw any definitive conclusions from these numbers due to the concomitant differences in recording conditions that were used in the different laboratories.

Our computer simulations provide further mechanistic insight into Hcn2/SkM1-based biological pacing following CPC-mediated delivery. These simulations suggest that transplantation of Hcn2/SkM1-expressing CPCs to working myocardium may be insufficient to generate stable pacemaker function, given the relatively large number of CPCs required to generate stable pacemaker activity. This was a surprising finding, given the substantial *in vivo* pacing after transplantation of Hcn2-expressing MSCs to the left ventricle of AV-block dogs (Potapova et al., 2004; Plotnikov et al., 2007; Jun et al., 2012; Lu et al., 2013). It is therefore conceivable that additional factors are involved that contribute to Hcn2-based biological pacing which are not well recapitulated by our computer model. Alternatively, it could suggest that a higher expression levels of Hcn2 and SkM1 are needed for appropriate functionality. Nonetheless, biological pacing improved in the context of lowering *I*_K__1_ as compared to the original human ventricular cell model (Ten Tusscher et al., 2004; Ten Tusscher and Panfilov, 2006), which is in line with our previous work that demonstrated superior biological pacemaker activity after adenoviral gene transfer to the left bundle branch (lower *I*_K__1_ environment) vs. subepicardial working myocardium (higher *I*_K__1_) (Boink et al., 2013). This setting also allowed us to further investigate the mechanistic contribution of SkM1 to Hcn2-based biological pacing. The associated computer simulations illustrate that SkM1 can generate additional inward current during late phase 4 depolarization, thereby facilitating spontaneous activity. This represents a new mechanism of action that further supports the robustness of Hcn2/SkM1-based biological pacemaker activity and can act synergistically with the previously demonstrated hyperpolarization of action potential threshold (Boink et al., 2013).

In conclusion, the present work identified lentiviral transduction as the method of choice to manipulate CPCs for long-term biological pacemaker applications. In this respect, CPC-mediated delivery of Hcn2/Skm1 appears promising, but functional animal studies are still needed to fully characterize this approach and make head-to-head comparisons to related strategies that rely on viral gene transfer or hiPSC-CMs.

## Data Availability Statement

The raw data supporting the conclusions of this article will be made available by the authors, without undue reservation.

## Ethics Statement

The studies involving human participants were reviewed and approved by the Medical Ethics Committee of the Leiden University Medical Center. The patients/participants provided their written informed consent to participate in this study. The animal study was reviewed and approved by Animal Ethics Committee of Leiden University Medical Center.

## Author Contributions

AMV, AS, AOV, AT, GB, HT, LC, MG, RN, RW, and VC contributed to conception and design of the study. AMV and AOV performed the statistical analysis. AMV wrote the first draft of the manuscript. AOV, LC, and RW wrote sections of the manuscript. DG, HD, JW, KL, LC, MK, MM-R, and RN contributed to data acquisition. All authors contributed to manuscript revision, read and approved the submitted version.

## Conflict of Interest

HT and GB report ownership interest in PacingCure B.V. The remaining authors declare that the research was conducted in the absence of any commercial or financial relationships that could be construed as a potential conflict of interest.
